# Macropinocytosis is decreased in diabetic mouse macrophages and is regulated by AMPK

**DOI:** 10.1186/1471-2172-9-42

**Published:** 2008-07-30

**Authors:** Christopher B Guest, Kenneth S Chakour, Gregory G Freund

**Affiliations:** 1 Division of Nutritional Sciences, University of Illinois at Urbana-Champaign, Urbana, USA; 2 Pathology, College of Medicine, University of Illinois at Urbana-Champaign, Urbana, USA

## Abstract

**Background:**

Macrophages (MΦs) utilize macropinocytosis to integrate immune and metabolic signals in order to initiate an effective immune response. Diabetes is characterized by metabolic abnormalities and altered immune function. Here we examine the influence of diabetes on macropinocytosis in primary mouse macrophages and in an *in vitro *diabetes model.

**Results:**

The data demonstrate that peritoneal MΦs from diabetic (*db/db*) mice had reduced macropinocytosis when compared to MΦs from non-diabetic (*db/+*) mice. Additionally, MΦs cultured in hyperglycemic conditions were less adept at macropinocytosis than those cultured in low glucose. Notably, AMP-activated protein kinase (AMPK) activity was decreased in MΦs cultured in hyperglycemic conditions. Activation of AMPK with leptin or 5-aminoimidazole-4-carboxamide-1-β-riboside (AICAR) increased macropinocytosis and inhibition of AMPK with compound C decreased macropinocytosis.

**Conclusion:**

Taken together, these findings indicate that MΦs from diabetic mice have decreased macropinocytosis. This decrease appears dependent on reduced AMPK activity. These results demonstrate a previously unrealized role for AMPK in MΦs and suggest that increasing AMPK activity in diabetic MΦs could improve innate immunity and decrease susceptibility to infection.

## Background

MΦs are critical mediators of various immune functions. One of the most important actions the MΦ plays is coordinating innate and adaptive immunity. To do this, MΦs must integrate signals from the local microenvironment with signals from the entire organism to assume an appropriate phenotype [1]. One of the ways MΦs do this is through macropinocytosis or 'drinking' large amounts of extracellular fluid. Macropinocytosis is characterized by the uptake of fluid through relatively large vacuoles (up to 5 microns) [2,3]. This process is similar to phagocytosis in a number of ways including the formation of an actin-rich ruffle with a structure similar to a pseudopodia and PI3-kinase dependent rearrangement of the plasma membrane [2,3]. However, these processes differ in some ways [2,3]. One of these differences is that phagocytosis utilizes ligand specific receptors while macropinocytosis is relatively non-specific allowing for a rapid unsaturable sampling of the heterogenous surrounding fluid including nutrients and pathogens. This fluid is concentrated and acidified in lysosomes, which usually destroy any pathogens contained within. The peptides are then processed and, in the case of professional antigen presenting cells, these peptides are shuttled back to the cell membrane in the context of MHC-II and can participate in the activation of T-cells [4]. Thus, macropinocytosis acts as a bridge between innate and adaptive immunity.

In the last decade, our understanding of how metabolism and immunity interact has grown a great deal. These complex systems share numerous components that are critical to ensure the fitness of an organism. One of the best examples of this interdependence is seen in diabetes. Diabetes is characterized by an inability to efficiently utilize glucose. As a result, those with diabetes are plagued by pathological complications including chronic inflammation [5-7] and a susceptibility to infectious pathogens including *Staphylococcus aureus*, *Streptococcus pneumonia *and *Mycobacterium tuberculosis *[8]. Another important modulator that is altered in diabetes is leptin. Leptin is a 16 kDa protein that is primarily secreted by adipose tissue and is structurally similar to the long-chain helical cytokine family including IL-6 [9]. It is recognized as an indicator of whole body energy levels and acts as a regulator of satiety in the hypothalamus [10]. However, studies with mice lacking a functional leptin system display a wide range of defects including impaired wound healing [11,12], increased uptake of modified LDL [13] and decreased phagocytosis of *Klebsiella pneumoniae *[14] indicating that leptin plays a significant function in immunity. Notably, several studies have shown that leptin activates AMPK in a number of cell types outside the central nervous system [15,16]. AMPK is well known as a master regulator of intracellular energy status, but recent work has also alluded to a roll as a key regulator of immune cell function including cytokine secretion [17,18] and chemotaxis [19]. In addition, activation of AMPK has been shown to improve some of the symptoms of diabetes [20]. Given the alterations to the immune system found in diabetes, we wanted to see if MΦs from diabetic mice had decreased macropinocytosis and, if so, determine which factors contributed to this alteration. Importantly, we found that macropinocytosis was decreased in MΦs cultured in hyperglycemic conditions and indicate that this decrease was caused by decreased AMPK activation.

## Results and discussion

### Peritoneal MΦs from diabetic (db/db) mice and MΦs cultured in diabetic conditions have decreased macropinocytosis

**Figure 1:**
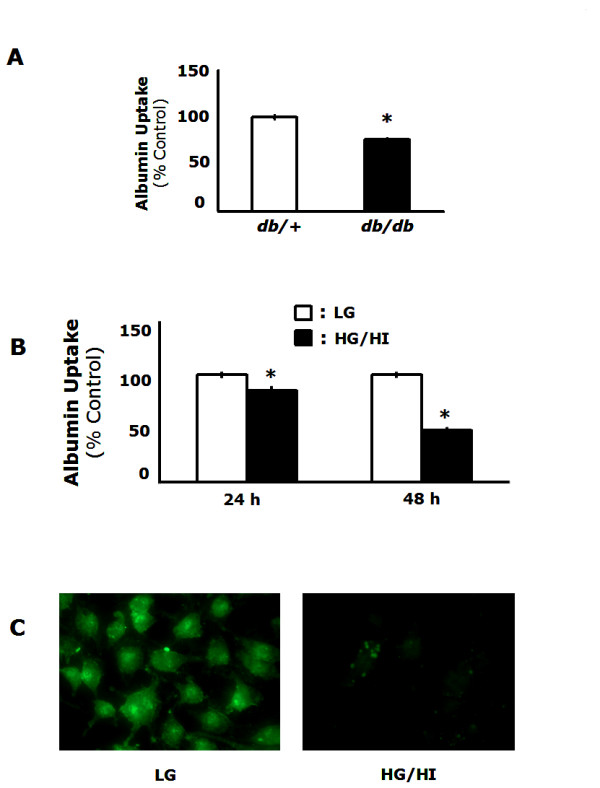
**Peritoneal MΦs from *db/db *mice and MΦs cultured in diabetic conditions have decreased macropinocytosis**. *A*, peritoneal MΦs were isolated from non-diabetic (*db/+*) and diabetic (*db/db*) and FITC-albumin uptake was measured by flow cytometry.*B*, MΦs were cultured in high glucose/high insulin conditions for 24 h or 48 h. FITC-albumin uptake was measured by flow cytometry. *C*, MΦs were cultured for 48 h in high glucose/high insulin conditions and FITC-albumin uptake was visualized by fluorescent microscopy. Results are representative of at least three independent experiments ± SEM. *: p < 0.05 from low glucose controls.

To determine if MΦ macropinocytosis was affected by diabetes, peritoneal MΦs were isolated from diabetic *db/db *mice and from non-diabetic *db/+ *mice and macropinocytosis was tested. Figure 1A demonstrates that peritoneal MΦs from *db/db *mice displayed diminished macropinocytosis when compared to MΦs from db/+ controls (p < 0.001). To determine which components of the diabetic milieu decreased macropinocytosis, the mouse MΦ cell line, RAW 264.7, was cultured for 24 h and 48 h in low glucose (1 g/L) and high glucose (4.5 g/L)/high insulin (1 nM) conditions observed in *db/db *mice [13]. MΦs cultured for 24 h in low glucose conditions had a 15% increase in the uptake of FITC-albumin when compared to cells cultured in high glucose/high insulin conditions (p = 0.02) (Figure 1B). When the duration of the culture was extended to 48 h, the difference increased to 51% (p < 0.001) (Figure 1B). This difference is further highlighted in Figure 1C by fluorescent microscopy. Taken together, these findings indicate that endocytosis of FITC-albumin by MΦs is decreased in two models of type 2 diabetes.

### Macropinocytosis is the fluid-phase endocytic process decreased in MΦs cultured in diabetic conditions

**Figure 2:**
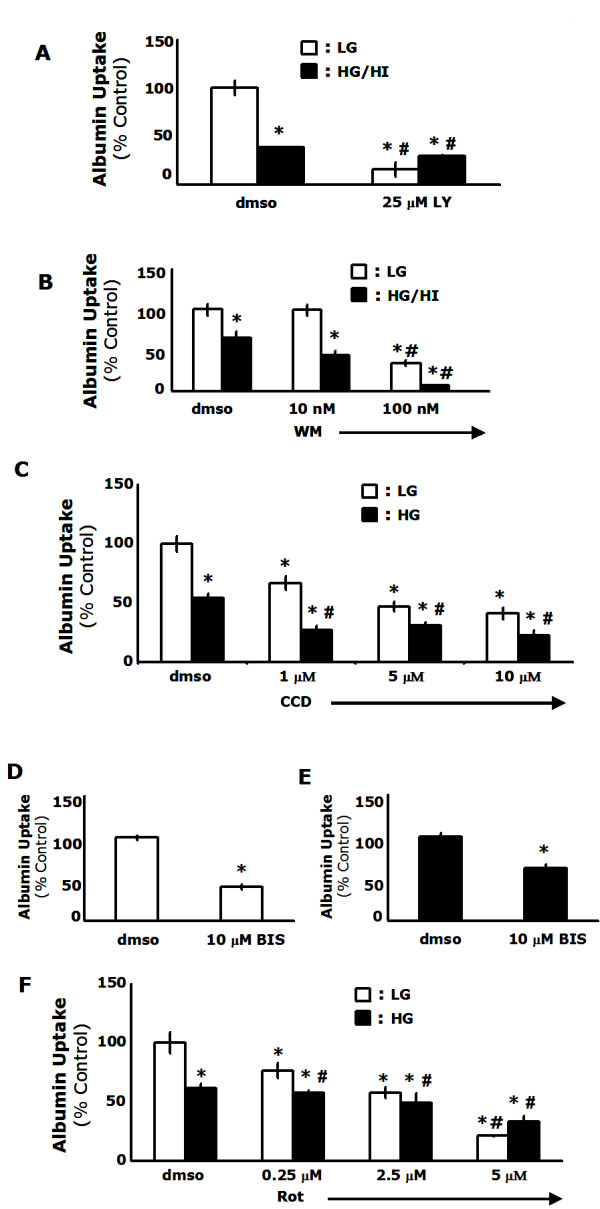
**Uptake of FITC-albumin is dependent on PI3-kinase, actin polymerization, and PKC-δ**. *A-B*, For PI3-kinase inhibition studies, MΦs were cultured in diabetic or non-diabetic conditions for 48 h and treated with 25 μM LY294002 or with wortmannin (10 nM or 100 nM) 15 m prior to uptake experiments. FITC-albumin uptake was measured by flow cytometry. *C*, MΦs were cultured in diabetic or non-diabetic conditions for 48 h and actin polymerization was inhibited by the addition of 1–10 μM of cytochalasin D 15 m prior to uptake studies. FITC-albumin uptake was measured by flow cytometry. *D-F*, For PKC inhibition studies MΦs were cultured in diabetic or non-diabetic conditions for 48 h and treated with 10 μM bisindolylmaleimide or 0.25–5 μM rottlerin 15 m prior to uptake experiments. FITC-albumin uptake was measured by flow cytometry. Results are representative of three independent experiments ± SEM. *: p < 0.05 from low glucose controls, *#: p < 0.05 from high glucose controls.

MΦs primarily utilize two pathways of fluid-phase endocytosis-macropinocytosis and micropinocytosis. Since macropinocytosis has been shown to require PI3-kinase [2], actin polymerization [3] and the PKC pathway [21], and micropinocytosis does not, we conducted experiments to determine the influence of these elements on the uptake of FITC-albumin. When cells were treated with the PI3-kinase inhibitor LY 294002, albumin uptake was reduced by 84% in cells cultured in low glucose conditions compared to untreated controls (p < 0.001). Similarly, uptake was significantly reduced in cells cultured in diabetic conditions (p = 0.002) (Figure 2A). In order to confirm this finding, we cultured cells in both low glucose and diabetic conditions and treated them with various concentrations of wortmannin, an alternate PI3-kinase inhibitor that works through a different mechanism [22]. Figure 2B indicates that treatment with 100 nM wortmannin as previously described [23] significantly reduced uptake in both low glucose (65%; p = 0.001) and diabetic conditions (89%; p = 0.001). Taken together, these data indicate that the uptake of FITC-albumin is PI3-kinase dependent. Next, we wanted to test whether actin polymerization regulated the uptake of FITC-albumin. Figure 2C shows that the uptake of FITC-albumin was inhibited by cytochalasin D in both low glucose (64%; p = 0.001 at 10 μM) and diabetic conditions (59%; p = 0.003 at 10 μM) in a dose dependent manner. PKCs have been implicated as key regulatory elements in macropinocytosis, particularly PKC*δ *[21]. When cells were treated with a pan-specific PKC inhibitor, bisindolylmaleimide, FITC-albumin uptake was reduced by 59% in cells cultured in low glucose conditions (p < 0.001) (Figure 2D). Similarly, uptake was significantly reduced in cells cultured in diabetic conditions (p = 0.003) (Figure 2E). In order to confirm this finding and determine which PKC isoform was the dominant regulator, we cultured cells in both low glucose and diabetic conditions and treated them with various concentrations of rottlerin, a specific PKC*δ *inhibitor [24]. Figure 2F indicates that treatment with rottlerin significantly reduced uptake in both low glucose (79%; p < 0.001 at 5 μM) and diabetic conditions (47%; p = 0.006 at 5 μM) in a dose dependent manner. These data suggest that the fluid-phase uptake of FITC-albumin is regulated by the PKC pathway and that PKC*δ *is the principal isoform. In summation, these results indicate that macropinocytosis is the fluid-phase endocytic process that is decreased in MΦs cultured in diabetic conditions.

### MΦs cultured in chronic hyperglycemic conditions have decreased macropinocytosis when compared to control cells

**Figure 3:**
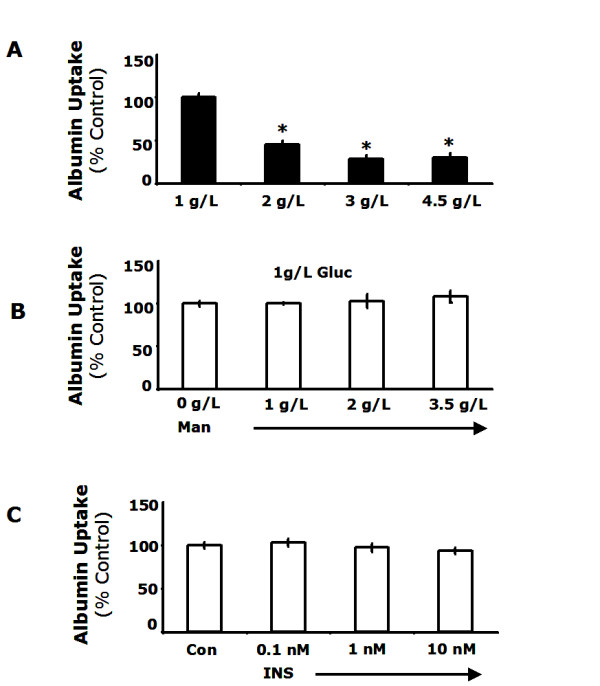
**Hyperglycemia reduced macropinocytosis while neither osmotic stress nor high insulin altered uptake**. *A*, MΦs were cultured for 48 h at the glucose concentrations indicated and FITC-albumin uptake was measured by flow cytometry. *B*, MΦs were cultured for 48 h in low glucose with mannitol at the concentrations indicated. FITC-albumin uptake was measured by flow cytometry. *C*, MΦs were cultured for 48 h in low glucose with insulin at the concentrations indicated. FITC-albumin uptake was measured by flow cytometry. Results are representative of three independent experiments ± SEM. *: p < 0.05 from low glucose controls.

In order to determine which factors were responsible for this difference in uptake, we tested whether high glucose, osmotic stress, or high insulin altered FITC-albumin uptake. Cells were cultured at various concentrations of glucose, mannitol or insulin for 48 h and FITC-albumin uptake was measured. FITC-albumin uptake decreased as the concentration of glucose increased. Uptake was reduced by 55% in cells cultured at 2 g/L glucose (p < 0.001) and 70% at either 3 g/L glucose or 4.5 g/L glucose (p < 0.001) (Figure 3A). Next, we tested whether this change was caused by osmotic stress using cells cultured in 1 g/L glucose with various concentrations of mannitol added to increase osmolarity to the levels seen in the high glucose experiments. The addition of mannitol had no discernible effect on FITC-albumin uptake even at the highest concentration tested (p = 0.28) (Figure 3B). Finally, we examined the effect of insulin on FITC-albumin uptake by culturing cells at various concentrations of insulin. Insulin failed to alter FITC-albumin uptake even at the highest concentration tested (p = 0.27) (Figure 3C). Taken together these findings indicate that macropinocytosis was decreased by culture in high glucose conditions and neither insulin nor osmotic stress significantly alter uptake.

### AMP-activated protein kinase activity is decreased in MΦs cultured in diabetic conditions and regulates macropinocytosis

**Figure 4:**
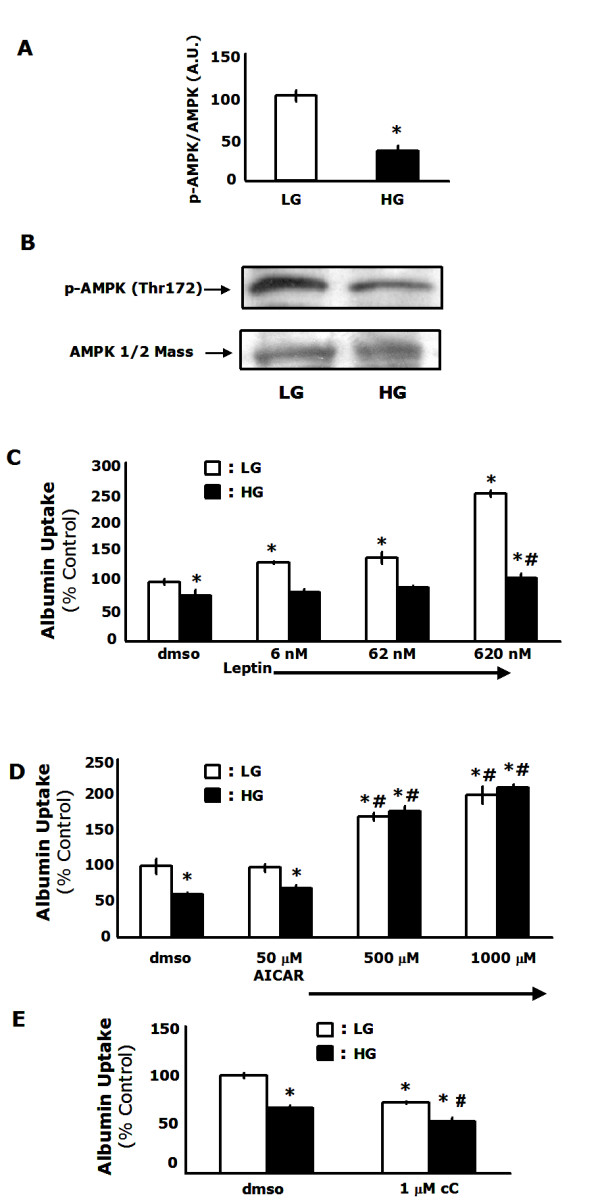
**AMP-activated protein kinase activity is decreased in MΦs cultured in diabetic conditions and regulates macropinocytosis**. *A-B*, MΦs were cultured for 48 h in low glucose or high glucose and α2 AMPK activity was measured by Western analysis. *C*, MΦs were cultured in low glucose or high glucose conditions with leptin at the concentrations indicated. FITC-albumin uptake was measured by flow cytometry. *D*, MΦs were cultured in low glucose or high glucose conditions with AICAR at the concentrations indicated. FITC-albumin uptake was measured by flow cytometry. *E*, MΦs were cultured in low glucose or high glucose conditions with 1 μM compound C. FITC-albumin uptake was measured by flow cytometry. Results are representative of three independent experiments ± SEM. *: p < 0.05 from low glucose controls, *#: p < 0.05 from high glucose controls.

AMPK is well known as a master regulator of intracellular energy metabolism [25]. Activation of AMPK has been found to increase glucose uptake in skeletal muscle [26] and alleviate some of the complications associated with diabetes [27-29]. Recently, AMPK has been found to regulate primary immune cell functions like chemotaxis [19], cytokine secretion [17,18] and uptake of *Candida albicans *[30]. In order to determine if AMPK played a role in the decreased macropinocytosis observed in MΦs cultured in diabetic conditions, we examined the phosphorylation state of threonine 172 on the α2 subunit of AMPK which has been shown to be crucial in the activation of AMPK [31]. AMPK activation was increased by 65% in cells cultured in low glucose conditions when compared to cells cultured in diabetic conditions (p < 0.001) (Figure 4A–B). This finding supports previous research indicating that AMPK is activated by low glucose conditions [32,33] and decreased in diabetic mice [34]. Next, we examined the role of AMPK in macropinocytosis by using the AMPK activator, leptin [15]. MΦs cultured in low glucose conditions demonstrated a dose dependent increase in macropinocytosis when treated with leptin ranging from 32% for 6 nM (p < 0.001) to 147% for 620 nM (p < 0.001) when compared to controls (Figure 4C). MΦs cultured in diabetic conditions also showed a dose dependent response to leptin treatment, although it was less pronounced, with uptake being significantly increased at the highest concentration of leptin tested (38%; p = 0.04) (Figure 4C). This attenuated response suggests a state of cytokine resistance. Cytokine resistance is a normal physiological counter-regulatory mechanism that decreases the potency of cytokines or other growth factors after repeated stimulation. However, chronic over-stimulation, as seen in diseases like diabetes, can lead to over-expression of suppressor-of-cytokine-signaling (SOCS) proteins which can inhibit off target receptors leading to immune or metabolic dysfunction [35]. We and others have reported that SOCS proteins are upregulated in diabetic MΦs [36] and inhibit leptin mediated activation of AMPK [37]. In order to further investigate this finding, we utilized a classical activator of AMPK, AICAR, which acts downstream of SOCS. AICAR is a cell permeable activator of AMPK that functions as a mimetic of AMP to facilitate phosphorylation by LKB1 [38] or CaMKK on the α2 subunit of AMPK [39]. MΦs cultured in low glucose exhibited a 100% increase in macropinocytosis when treated with 1 mM AICAR (p = 0.002). Macrophages cultured in high glucose exhibited an increase of 244% when treated with 1 mM AICAR (p < 0.001) compared to untreated high glucose controls (Figure 4D). Interestingly, this finding may indicate that other types of immunological and metabolic abnormalities caused by cytokine resistance can be attenuated or reversed by activating AMPK by pathways independent of receptor mediated activation. Finally, in order to further test whether AMPK was a critical regulator of macropinocytosis, we used the AMPK inhibitor, compound C [40,41]. Chronic treatment with compound C significantly decreased macropinocytosis in MΦs cultured in either low glucose (28%; p < 0.001) or diabetic conditions (27%; p = 0.017) (Figure 4E). Importantly, FITC-albumin uptake was not completely abolished by treatment with compound C at the concentration used, indicating that there are other important factors that regulate macropinocytosis. Taken together, these results indicate that the decreased macropinocytosis observed in MΦs cultured in diabetic conditions is caused by decreased AMPK activation.

## Conclusion

MΦs must be capable of adapting to diverse immune and metabolic environments ranging from the hypoxic, nutrient poor surroundings of a necrotic tumor to the well oxygenated nutrient rich environment of the lung. In order to provide the energy necessary to perform their immune functions, MΦs either utilize glycolysis or oxidative phosphorylation. Work by Odegaard et al., shows that this switch is a critical determinant of macrophage phenotype and function [42]. Additionally, they showed that disruption of this phenotypic switch may be an important component of insulin resistance and hyperglycemia.

**Figure 5:**
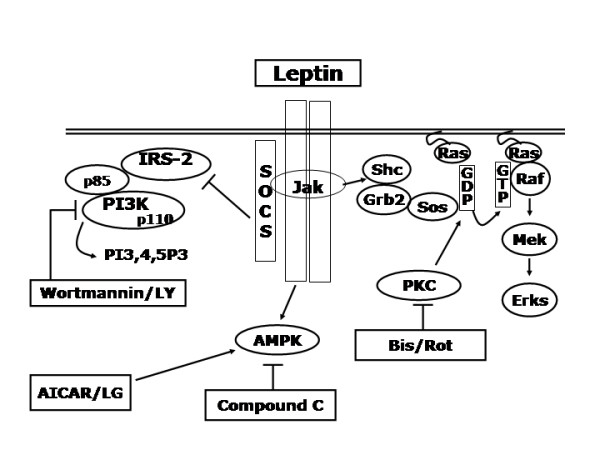
**Summary schematic of the pathways regulating macropinocytosis**. AMPK, AMP-activated protein kinase, ERK, extracellular-signal-regulated kinase, PI3K, phosphoinositide 3-kinase, PKC, protein kinase C, SOCS, suppressor of cytokine signaling, IRS-2, insulin receptor substrate 2, Jak, janus kinase.

Macropinocytosis is a highly energetic process requiring extensive restructuring of the plasma membrane and the coordination several pathways including PI3-kinase and PKCs. One of the key enzymes regulating the transition from oxidative phosphorylation to glycolysis is AMPK. Interestingly, since PI3-kinase and PKCs are involved in numerous other endocrine and immune roles, AMPK may be a critical mediator of these other functions. Furthermore, our findings present new evidence that demonstrate a novel role for AMPK in regulating an important aspect of innate immunity and indicate a central role for AMPK in determining macrophage phenotype. In addition, we also show that diabetic conditions, particularly hyperglycemia, can impair macropinocytosis through decreased AMPK activation. Further work needs to be done to determine the role of AMPK in the regulation of macrophage phenotype and function in health and disease and how AMPK interacts with other known regulators of macrophage function (Figure 5).

## Materials and methods

### Materials

The mouse macrophage like cell line, RAW 264.7, was purchased from American Type Culture Collection (Rockville, MD). All cell culture reagents and chemicals were purchased from Sigma (St. Louis, MO) except as noted below. FBS (0.05 ng/mL, 0.48 EU/mL endotoxin) and Leptin were purchased from Atlanta Biologicals (Norcross, GA). LY 294002 (Cat# 440202), rottlerin (Cat# 557370), 5-Aminoimidazole-4-carboxamide-1-β-riboside (AICAR) (Cat# 123040), 6-[4-(2-Piperidin-1-yl-ethoxy)-phenyl)]-3-pyridin-4-yl-pyrrazolo [1,5-a]-pyrimidine (compound C) (Cat# 171260) and bisindolylmaleimide (Cat# 203293) were purchased from Calbiochem (La Jolla, CA). Propidium iodide was purchased from Molecular Probes (Eugene, OR). Anti-AMPKα 1/2 (Cat# sc-25792), anti-phoso AMPK (Threonine 172) (Cat# sc-33524-R) and Culture Micro Slides (sc-24978) were purchased from Santa Cruz Biotechnology (Santa Cruz, CA). Fluormount G was purchased from Southern Biotechnology.

### Animals

All animal care and use was conducted in accordance with the Guide for the Care and Use of Laboratory Animals (NRC). 8- to 12-week-old B6.Cg-*M*+/+*Lepr*^db ^(*db*/+) and B6.Cg-+*Lepr*^db^/+*Lepr*^db ^(*db/db*) were bred in house from mice purchased from The Jackson Laboratories (Bar Harbor, Maine). Mice were housed in standard shoebox cages and allowed pelleted food (NIH 5K52; LabDiet, Purina Mills Inc., Brentwood, MO) and water *ad libitum *in a temperature (72°C) and humidity (45–55%) controlled environment with a 12/12-h dark-light cycle (7:00 a.m. – 7:00 p.m.).

### Peritoneal Macrophage Isolation

Mice were sacrificed by CO_2 _asphyxiation and peritoneal fluid was collected by lavaging the peritoneum twice with 5 ml ice cold low glucose growth media (glucose-free RPMI 1640 media supplemented with 10% FBS, 1 g/L glucose, 2 g/L sodium bicarbonate, 110 mg/L sodium pyruvate, 62.1 mg/L penicillin and 100 mg/L streptomycin, 10 mM HEPES pH 7.4), followed immediately by analysis or use in *ex vivo *experiments. For *ex vivo *experiments, cells were plated according to the following procedure. Peritoneal fluid was centrifuged and the resulting pellet resuspended in 5 ml of red blood cell lysis buffer (142 mM NaCl, 118 mM NaEDTA, 1 mM KHCO_3 _pH 7.4) at room temperature for 4 minutes. An equal volume of cold low glucose growth media was added followed by cell pelleting and resuspension in 37°C low glucose growth media. Cells were counted with the use of a hemocytometer and plated in culture dishes at 5 × 10^5 ^cells/ml in low glucose growth media. After 30 min, plates were washed twice to remove non-adherent cells, resulting in approximately 80% pure macrophages, as previously confirmed by CD11b staining and morphology [13]. Immediately following plating selection, peritoneal macrophages were used for uptake experiments.

### Cell Culture/Insulin and Glucose Treatment

Raw 264.7 cells were grown in RPMI growth media (2 g/L glucose) as previously described [24]. For glucose and mannitol treatments, cells were resuspended at 2.5 × 10^5 ^cells/ml in low glucose growth media (1 g/L glucose) or supplemented with additional glucose or mannitol at the concentrations indicated and grown for 48 h. For chronic insulin treatments, cells were treated as above with or without the addition of 1 nm insulin or at the concentrations indicated for 48 h. Cell viability was at least 80% as determined by PI staining.

### Macropinocytosis

Quantitative analysis of macropinocytosis was performed as described [13,43,44], with minor modifications. Cells (2.5 × 10^5^) were incubated with 5 μg/mL FITC-albumin at 37°C for 4 h. Macropinocytosis was stopped by 3 washes in ice-cold wash buffer. Gates were set to exclude PI positive cells. For PI3-kinase inhibition experiments, 25 μM LY294002 or wortmannin (10 nM or 100 nM) was added 15 m prior to uptake experiments. For inhibition of actin polymerization studies, 1–10 μM of cytochalasin D was added 15 m prior to uptake studies. For PKC inhibition studies, 10 μM bisindolylmaleimide or 0.25–5 μM rottlerin was added 15 m prior to uptake experiments. For leptin experiments, 6–620 nM leptin was added 48 h prior to uptake experiments. For AICAR experiments, 50–1000 μM AICAR was added 48 h prior to uptake experiments. For compound C experiments, 1 μM compound C was added 48 h prior to uptake experiments. For fluorescent imaging studies cell grown in Culture Micro Slides and treated as indicated. Cells were fixed with 10% formalin for 15 m and coverslipped with Fluormount G. Cells were then visualized using the FITC filter.

### Western Analysis

Macrophages were lysed in ice-cold lysis buffer and proteins resolved by SDS-PAGE (25 μg/lane) under reducing conditions in 4–20% gradient gels. After electrotransfer to nitrocellulose and resolution of proteins with ECL Plus Kit (Amersham) [36], bands where quantified by densitometry and analyzed using *Image J *(NIH), The numerical data reported in the 'results' section is based on the summary of all blots followed by statistical analysis.

### Statistical Analysis

Data are presented as mean ± SEM. Experimental data were analyzed either by the Student's t-test for comparison of means, or by ANOVA using Excel (Microsoft, Redmond WA). Statistical significance was denoted at p < 0.05.

## Authors' contributions

CBG conceived of the study, carried out flow cytometry studies and drafted the manuscript. KSC carried out flow cytometry experiments and analyzed data. GGF participated in the study design, data analysis and manuscript preparation. All authors have read and approve of the manuscript.
